# Antibacterial ability of different activated irrigation after root canal preparation: intratubular analyses

**DOI:** 10.1590/0103-6440202405883

**Published:** 2024-05-10

**Authors:** Cleber Keiti Nabeshima, Hector Caballero-Flores, Elisabete José Vicente, Giulio Gavini, Manoel Eduardo de Lima Machado

**Affiliations:** 1 Department of Restorative Dentistry, School of Dentistry, University of São Paulo, São Paulo, SP, Brazil; 2 Department of Microbiology, Institute of Biological Science, University of São Paulo, São Paulo, SP, Brazil

**Keywords:** Endodontics, disinfection, dentinal tubules, final irrigation, activation

## Abstract

This study assessed the intratubular antibacterial ability of different activated irrigations after chemical mechanical preparation. Seventy-two palatal root canals of upper molars were infected with Enterococcus faecalis for 4 weeks, and then initial bacterial collection from the main root canal was performed. The root canals were prepared by using a WaveOne Gold large (45/.05) and distributed into 6 groups according to the activation of the final irrigation: ultrasonic activation (UA), XP-Endo Finisher (25/.00), XP Clean (25/.02), EasyClean (25/.04) in reciprocating motion and continuous rotary motion (ECRot), and conventional irrigation. After final irrigation, another bacterial collection from the main root canal was performed, and the root was sectioned transversely in three-thirds and stained for analysis by confocal laser microscopy. Intratubular bacteria were collected through dentin powder and plated for bacterial viability analysis. Intergroup and intragroup comparisons were performed by using analysis of variance and repeated measures analysis of variance, respectively, both at 5% significance. ECRot had higher antibacterial ability than UA (p<0.05), and both were superior to the other groups (p<0.05) in both methodologies. It can be concluded that activation of final irrigation enhances the disinfection of the root canal system, and activators have different efficacies

## Resumo

Este estudo avaliou a capacidade antibacteriana intratubular de diferentes irrigações ativadas após o preparo químico-mecânico. Setenta e dois canais palatinos de molares superiores foram contaminados com *Enterococcus faecalis* por 4 semanas, e então a coleta bacteriana inicial do canal principal foi realizada. Os canais foram preparados com WaveOne Gold large (45/.05) e distribuídos em 6 grupos de acordo com a ativação da irrigação final: ativação ultrassônica (UA), XP-Endo Finisher (25/.00), XP Clean (25/.02), EasyClean (25/.04) em movimento reciprocante e em movimento rotatório contínuo (ECRot) e irrigação convencional. Após a irrigação final, outra coleta bacteriana do canal principal foi realizada, e a raiz foi seccionada transversalmente em três terços e corada para análise por microscopia laser confocal. Bactérias intra tubulares foram coletadas através de pó de dentina e cultivadas para análise de viabilidade bacteriana. As comparações entre grupos e intragrupos foram realizadas usando análise de variância e análise de variância de medidas repetidas, respectivamente, ambas com 5% de significância. ECRot apresentou maior capacidade antibacteriana que UA (p<0,05), e ambos foram superiores aos demais grupos (p<0,05) em ambas as metodologias. Pode-se concluir que a ativação da irrigação final melhora a desinfecção do sistema de canais radiculares, e os ativadores têm eficácias diferentes.

## Introduction

A recent systematic review showed that more than half of the world's adult population has at least one tooth with apical periodontitis ^1^. This pathological manifestation is an inflammatory reaction caused by bacteria and endotoxins from the root canal system and has been identified as a source of pro-inflammatory substances such as C-reactive protein, interleukin 6, asymmetric dimethylarginine, and C3 complement fragment levels that are released into the circulatory system by damaging other body tissues ^2^. Although the success rate for primary endodontic treatment is high, persistent cases are still present ^3^. The literature has not yet been able to establish the minimum number of bacteria for the development and maintenance of periapical periodontitis, but additional strategies to enhance the disinfection of the root canal system could lead to better conditions for periapical healing.

Chemical mechanical preparation has been efficient for disinfecting the main canal at a rate of up to 100% bacterial reduction ^4^, but the bacteria present in intricate areas such as isthmuses, ramifications, recesses, and dentinal tubules may not be affected by the antimicrobial intracanal procedures and are often the cause of persistent disease even in well-treated teeth ^5^. In addition, persistent disease is associated with bacteria located in the apical segment of the root canal ^5^. Conventional final irrigation with a needle and syringe has been used for many years but it presents limitations as it is not able to reach the depth of the dentinal tubules ^6^, in addition, it does not deliver the irrigant properly throughout the root canal and lateral canals ^7^.

Ultrasonic activation (UA) has been proposed to improve the cleaning of the root canal and enhance the antibacterial ability in the dentinal tubules ^8^ ^,^ ^9^, but new instruments promoting mechanical agitation of irrigants have been developed. XP-Endo Finisher (FKG Dentaire, La Chaux-de-Fonds, Switzerland) is a 25/.00 rotary instrument made of a heat-treated nickel-titanium alloy called MaxWire, which changes its shape when subjected to a temperature of 35°C, expanding the range of the instrument over the root canal walls ^10^. XP Clean (MK Life, Porto Alegre, Brazil) is a 25/.02 wavy-shaped rotary instrument made of heat-treated nickel-titanium alloy, which when in rotation, can reach a greater area of the root canal. Lastly, Easy Clean (Easy Equipamentos Odontológicos, Belo Horizonte, Brazil) is a 25/.04 plastic instrument to be used in continuous rotation or reciprocating motion for the removal of dentin debris ^11^. Evaluations of these agitators target the main canal, isthmus, and lateral canals ^7^ ^,^ ^12^ ^,^ ^13^. Studies regarding intratubular antimicrobial ability are scarce, and they evaluate the final irrigation no considering the chemical mechanical preparation ^9^.

In light of the above, this study aimed to assess the intratubular antibacterial ability of different activated irrigations after chemical mechanical preparation by using two methodological analyses: culture method and confocal laser microscopy. The null hypothesis was that the type of final irrigation activation after root canal instrumentation does not influence the bacterial reduction in dentinal tubules.

## Material and Methods

### Sample calculation

The sample size calculation was performed with G*Power software 3.1 (Franz Faul, Unisersität Kiel, Germany), and analysis of variance was applied to data from a pilot test using 5 samples per group. The area of the damaged bacteria after treatments was expressed as a percentage and as the mean and standard deviation (Table 1). The effect size was 2.79 for the coronal third, 2.69 for the middle third, and 4.47 for the apical third, with an alpha error of 0.05 and a beta power of 0.8, resulting in a total of 12 samples per group.

**Table 1:** Mean ± standard deviation of the percentage area of the damaged bacteria after treatments: data from the pilot study for sample calculation.

Groups	Coronal	Medium	Apical
UA	44.97 ± 1.25	44.87 ± 1.67	44.68 ± 1.13
XPF	35.75 ± 3.10	34.71 ± 3.29	34.32 ± 1.13
XPC	37.82 ± 1.21	37.08 ± 1.08	36.45 ±1.20
ECRec	37.56 ± 0.68	37.10 ± 1.00	36.84 ± 0.58
ECRot	52.45 ± 2.32	52.20 ± 2.17	52.16 ± 2.01
C	24.16 ± 1.61	23.48 ± 2.03	22.99 ± 1.68

UA - ultrasonic activation, XPF - XP-Endo Finisher, XPC - XP Clean, ECRec - EasyClean in reciprocating motion, ECRot - EasyClean in continuous rotary motion, C - conventional.

### Selection and standardization of specimens

After approval by the local research ethics committee (CEP-FOUSP #3.065.715), 72 palatal roots of maxillary molars with a single, round, and straight canal were selected by periapical radiography in both mesiodistal and buccolingual directions. Teeth with palatal root canals smaller than 12 mm in length, calcified root canals, diameters wider than a #25 K-file, and with an open apex were replaced.

The palatal roots were removed and standardized at 12 mm in length, and the working length was set to 11 mm with a #15 K-file (Dentsply Sirona, Ballaigues, Switzerland). After confirming the patency with a #10 K- file, the diameter of the root canals was standardized with WaveOne Gold Primary file (Dentsply Sirona) by being introduced into the root canal using 3 pecking motions in the apical direction. Then, the root canal was explored up to working length using a #15 K file. This kinematics was performed until reaching full working length.

The roots were immersed in 2.5% sodium hypochlorite solution (Fórmula & Ação, São Paulo, Brasil), followed by distilled water, 17% EDTA (Fórmula & Ação), and distilled water again, all in an ultrasonic bath for 4 minutes. Every 8 roots were vertically placed into a 24-well cell plate containing condensation silicone (Flex-sil, Technew, Rio de Janeiro, Brazil) before being sterilized with gamma radiation.

### Bacterial preparation

*Enterococcus faecalis* (ATCC 29212) in glycerol was defrosted, vortexed for 1 min, and plated on m-Enterococcus agar (Difco, Le Pont-de-Claix, RA, France) before being incubated in aerobiosis at 37°C for 48h. A growing colony was collected and vortexed in BHI broth (Difco) and incubated again at 37°C for 24h. Then, an aliquot of 100 µL was resuspended in BHI broth and standardized at McFarland scale 1 (3x10^8^ cells mL^-1^) after 4 hours of cultivation (exponential phase).

### Contamination of specimens

After sterilization, the roots were removed from the cell plate, immersed in BHI broth, and ultrasonicated for 15 minutes before being taken individually into propylene microtubes containing 1 mL of suspension of *Enterococcus faecalis*. The microtubes were centrifuged for two cycles at 1400 g, 2000 g, 3600 g, and 5600 g for 5 minutes each, with the broth being exchanged for a new bacterial suspension at each centrifugation ^14^. Next, the roots were incubated in bacterial suspension at 37°C for 4 weeks in aerobiosis with BHI broth renewed every 24 hours being the centrifugation cycles repeated on 48th and 96th hours.

### Initial collection

The external root surface of the specimens was scraped with a scalpel blade (Descarpack, São Paulo, SP, Brazil) and cleaning was carried out using a cotton swab (Cral Absorve, Cotia, SP, Brazil) with 3% hydrogen peroxide, followed by 2.5% sodium hypochlorite and inactivation with 5% sodium thiosulfate (Fórmula & Ação). Disinfection was confirmed by applying a sterile #80 paper point to the external surface, and subsequently incubating in BHI broth at 37°C for 48h.

The roots were replaced into the plate containing condensation silicone before being filled with saline solution for initial bacterial collection by introducing three paper points (WaveOne Gold Primary, Dentsply Sirona) into the root canal for 1 minute each and then immersing them into 1 mL of saline solution followed by agitation in vortex for 1 minute. Serial dilution was performed and plated in triplicate on m-Enterococcus agar and incubated at 37°C for 48 hours followed by counting in CFU mL^-1^.

### Root canal preparation

The root canals were explored with a #25 K-file up to the working length, and the plate was placed in a thermal chamber at 37°C. The root canals were prepared by using a Wave One Gold Large file (Dentsply Sirona) in a reciprocating motion as previously described. Irrigation was performed with a total of 20 mL of 2.5% sodium hypochlorite solution by using a 29-gauge side-vented needle (Ultradent Products, South Jordan, USA) with in-and-out movement at 2 mm short of the working length. The total contact time of the irrigant with the root canal was standardized at 10 minutes.

### Distribution of groups

The roots were randomly distributed into six groups (n=12) according to the final irrigation as follows: ultrasonic activation (UA) with the insert (Irrisonic 20/.01, Helse Dental Technology, Santa Rosa de Viterbo, Brazil) mounted on an ultrasonic device (Gnatus, Ribeirão Preto, Brazil) at power 2 and located 1 mm short of the working length; XP-Endo Finisher (XPF) and XP Clean (XPC) files, both in continuous rotary motion (X-Smart Plus, Dentsply Sirona) at 800 rpm and torque of 1 Ncm with slow longitudinal movements of 7-8 mm amplitude up to the working length; EasyClean in reciprocating motion (ECRec) and EasyClean in continuous rotary motion (ECRot) at a speed of 1,000 rpm and torque of 1 Ncm (X-Smart Plus), both positioned at 1 mm short of the working length; and conventional irrigation (C) with syringe and a 29-gauge side-vented needle with in-and-out movement up to 1 mm short of working length.

A sequence of 2.5% sodium hypochlorite solution, 17% EDTA, and 2.5% sodium hypochlorite solution was used in 3 cycles of 2 ml each in all the groups. Irrigant activation was performed for 20 seconds between each cycle, totaling 1 minute per irrigant. All the irrigants used during preparation and final irrigation were heated to 37°C.

### The second collection from the main root canal

Inactivation with 2 mL of 5% sodium thiosulfate solution was performed for 5 minutes after final irrigation. Next, another bacterial sample from the main root canal was collected as previously described by using paper points WaveOne Gold Large and incubated in 500 µl of BHI broth at 37°C for 24 hours in aerobiosis before being plated on m-Enterococcus agar to verify the bacterial growth.

### Intratubular analysis by confocal laser microscopy

Apical and coronal slices of 1 mm each were removed from each sample using a fine steel disc at low speed, and then the root was sectioned transversely at 6 and 3 mm from the apex, resulting in three-thirds. All steps were carried out inside a laminar flow hood for aseptic conditions.

The samples were immersed in 17% EDTA solution for 1 minute and stained with the LIVE/DEAD BacLight Bacterial Viability Kit (Invitrogen, Carlsbad, CA, USA) for 20 minutes in the dark before washing in saline solution for 5 minutes. The upper surface of the samples was scanned in mosaic mode by using a confocal laser microscope (LSM 780 NLO, Carl Zeiss, Oberkochen, Deutschland) at wavelengths of 480/500 nm for SYTO 9 and 490/635 nm for propidium iodide stains with a 20x lens, 0.6x magnification and resolution of 1028 x 1028 pixels. The final image field was the center of the root canal up to a depth of 300 µm from the dentin wall. The green and red channels were separated by using FIJI ImageJ 1.53c software (Wayne Rasband, National Institutes of Health, USA) for the quantification of bacteria with intact and damaged cell walls, respectively (Figure 1). The layers of each channel were overlapped, and the staining was selected by using the threshold tool in automatic mode, with the marked area calculated in µm^2^. The percentage of area regarding damaged bacteria was calculated according to the following formula:

Percentage = $\frac{redarea}{redarea+greenarea}$

**igure 1:**
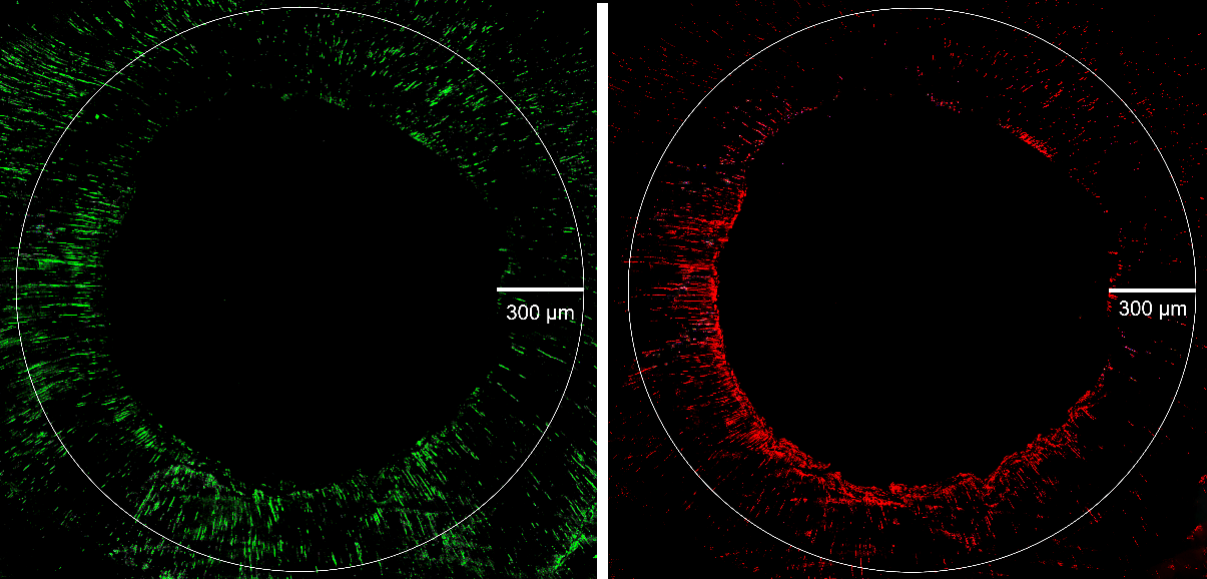
Green and red channels with overlapping layers for analysis of intratubular staining. Original magnification

### Intratubular bacterial count

After scanning, the intratubular bacteria were collected by using dentin powder and Peeso reamers (VDW, München, Germany) with diameters corresponding to the root thirds as follows: up to #4 for apical, #5 for middle and #6 for coronal thirds. The dentin powder was weighed and added to 1 mL of saline solution for serial dilution and plated in triplicate on m-Enterococcus agar before incubation at 37°C for 48 hours, followed by counting in CFU mg^-1^.

### Statistical analysis

All data were submitted for statistical analysis using Jamovi 2.3 software (Jamovi project, Sydney, Australia). Shapiro-Wilk's test was used to verify the distribution of normality. Intergroup comparisons were performed by using analysis of variance, and intragroup comparisons were performed by using repeated measures analysis of variance, both complemented by Tukey’s test. All statistical tests were performed at a significance level of 5%.

## Results

The initial collection showed a similar bacterial count between the groups (p>0.05), and all samples from the root canal lumen had no bacterial growth after root canal preparation and final irrigation throughout the 24 hours of incubation (Table 2).

**Figure 2:**
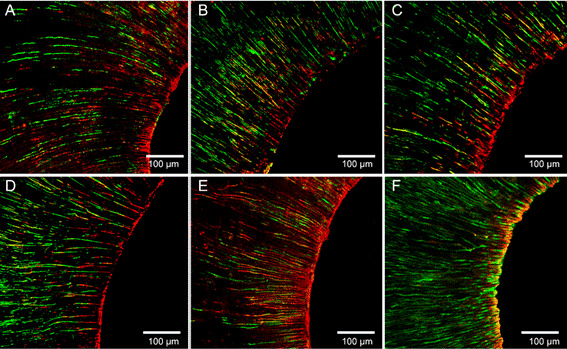
Representative image of the antibacterial action of different methods of final irrigation in dentinal tubules: UA (A), XPF (B), XPC (C), ECRec (D), ECRot (E), and conventional (F). Red, green, and yellow stains indicate the presence of damaged, intact, and both (damaged and intact overlapped) bacteria, respectively. Original magnification.

Culture and confocal laser microscopy analysis of intratubular bacteria showed that ECRot had fewer viable bacteria and a higher percentage of damaged bacteria than UA and that both had superior antibacterial ability than the other groups (p<0.05). Conventional irrigation had the highest number of viable bacteria and the lowest percentage of damaged bacteria (p<0.05; Figure 2).

The comparison between the root thirds showed that the number of intratubular bacteria was lower in the apical third than in the coronal and middle thirds in all groups (p<0.05), but the percentage of damaged bacteria was similar in the three root thirds when observed by confocal laser microscopy (p>0.05).

**Table 2:** Mean ± standard deviation of the bacterial count from the main root canal before and after chemical mechanical preparation (UFC mL^-1^) using different activators for final irrigation

Groups	Initial collection	After final irrigation
UA	1.07 x 10^7^ ± 8.45 x 10^5^	0 ± 0
XPF	1.05 x 10^7^ ± 4.89 x 10^5^	0 ± 0
XPC	1.05 x 10^7^ ± 6.13 x 10^5^	0 ± 0
ECRec	1.05 x 10^7^ ± 6.67 x 10^5^	0 ± 0
ECRot	1.02 x 10^7^ ± 5.22 x 10^5^	0 ± 0
C	1.06 x 10^7^ ± 4.88 x 10^5^	0 ± 0

UA - ultrasonic activation, XPF - XP-Endo Finisher, XPC - XP Clean, ECRec - EasyClean in reciprocating motion, ECRot - EasyClean in continuous rotary motion, C - conventional.

The culture method showing the number of viable bacteria and percentage of the area regarding damaged bacteria can be observed in Tables 3 and 4, respectively.

**Table 3:** Mean ± standard deviation of the bacterial count from dentin (UFC mg^-1^) in different thirds

Groups	Coronal	Medium	Apical ^*^
UA ^a^	4.78 x 10^5^ ± 2.41 x 10^4^	4.70 x 10^5^ ± 3.17 x 10^4^	4.31 x 10^5^ ± 1.06 x 10^4^
XPF ^b^	7.27 x 10^5^ ± 3.93 x 10^4^	7.16 x 10^5^ ± 4.15 x 10^4^	5.78 x 10^5^ ± 2.98 x 10^4^
XPC ^b^	7.28 x 10^5^ ± 3.44 x 10^4^	7.06 x 10^5^ ± 3.64 x 10^4^	5.47 x 10^5^ ± 1.85 x 10^4^
ECRec ^b^	7.26 x 10^5^ ± 5.38 x 10^4^	7.16 x 10^5^ ± 3.31 x 10^4^	5.56 x 10^5^ ± 1.86 x 10^4^
ECRot ^c^	3.71 x 10^5^ ± 1.02 x 10^4^	3.78 x 10^5^ ± 2.16 x 10^4^	3.54 x 10^5^ ± 7.43 x 10^3^
C ^d^	1.13 x 10^6^ ± 6.06 x 10^4^	1.09 x 10^6^ x 6.74 x 10^4^	9.73 x 10^5^ ± 5.55 x 10^4^

^a^ ^-d^ Different letters indicate differences between groups in all thirds, ANOVA/Tukey’s test (p<0.05). *Asterisks represent differences between thirds, repeated measures ANOVA/Tukey’s test (p<.0.05). UA - ultrasonic activation, XPF - XP-Endo Finisher, XPC - XP Clean, ECRec - EasyClean in reciprocating motion, ECRot - EasyClean in continuous rotary motion, C - conventional.

**Table 4:** Mean ± standard deviation of the percentage of area (%) regarding damaged bacteria in different thirds

Groups	Coronal	Medium	Apical
UA ^a^	44.20 ± 1.52	43.55 ± 2.37	43.32 ± 1.85
XPF ^b^	36.12 ± 2.13	35.39 ± 2.44	35.02 ± 1.42
XPC ^b^	37.17 ± 1.19	36.72 ± 1.13	36.49 ± 1.25
ECRec ^b^	37.44 ± 0.73	36.84 ± 0.93	36.61 ± 0.87
ECRot ^c^	52.86 ± 1.81	52.64 ± 1.55	52.29 ± 1.68
C ^d^	25.35 ± 1.79	24.80 ± 2.13	24.65 ± 2.45

^a^ ^-d^ Different letters indicate differences between groups in all thirds, ANOVA/Tukey’s test (p<0.05). UA - ultrasonic activation, XPF - XP Endo Finisher, XPC - XP Clean, ECRec - EasyClean in reciprocating motion, ECRot - EasyClean in continuous rotary motion, C - conventional.

## Discussion

Various types of irrigation have been explored for disinfection of the root canal system. This study assessed different activation methods after chemical mechanical preparation, in which it was demonstrated that the activation of the final irrigant potentiates the antibacterial action within the dentinal tubules and that different activators have different efficacies. Therefore, the null hypothesis was rejected for both methodologies.

The literature regarding root canal disinfection has used paper points for bacterial collection ^4^ ^,^ ^12^ ^,^ ^13^, however, this methodology is not capable of disrupting biofilm and collecting microorganisms from dentinal tubules. In the present essay, dentin collection using Peeso reamers was carried out targeting intratubular bacteria, and paper points were used in previous collections targeting the main canal as complementary information. The culture method using dentin collection has limitations as it does not have initial bacterial quantification to calculate the percentage of reduction. Therefore, the comparison between the groups is carried out only by a final number of bacteria still viable after treatment. Confocal laser microscopy is a method that quantifies intact and damaged bacteria by staining the bacterial genetic code in which it is possible to consider the total number of intratubular bacteria before treatment. However, unlike the culture method by uses dentin collection, it allows an analysis of multiple layers in a limited segment of the sample, then, it does not cover the entire root third. In this way, the two methods complement each other for a more robust intratubular analysis.

The initial collection from the main root canal showed that all groups had similar bacterial growth, which demonstrates the uniformity of contamination between them. The absence of bacterial growth in the main root canal after preparation and final irrigation implies the effectiveness of the mechanical action associated with the irrigant, as also observed by Fernandes et al. ^13^, who evaluated UA and ECRec. Nakamura et al. ^4^ observed the absence of bacterial growth in the main root canal using 5.25% NaOCl, and our results were similar using 2.5% NaOCl. In contrast, Carvalho et al. ^12^ showed positive cultures after root canal preparation with 2.5% NaOCl, but they evaluated oval root canals and did not use EDTA in the final irrigation. The use of a chelator (e.g., EDTA) is justified for the removal of the smear layer, which reduces the antibacterial action of the irrigant on dentinal tubules ^15^. In the present study, the root canals were standardized to a round shape before contamination, but anatomy is a factor to be considered as well, as surfaces not touched by the instrument are left after the preparation of the non-round root canals ^16^. Azim et al. ^9^ also did not find bacterial negativity after conventional final irrigation and use of XPF, although they did not evaluate the final irrigation associated with root canal preparation.

The comparison between the groups regarding intratubular bacteria had the same result in both methodologies. The difference between ECRot and ECRec demonstrates that the kinematics of these instruments interfere with the intratubular antibacterial ability, probably because continuous rotary motion exerts a greater force than reciprocating motion by increasing the penetration of the irrigant into the dentinal tubules. The superiority of ECRot over ECRec was also observed in a study evaluating the penetration of irrigants into lateral root canals ^7^, which corroborates our results.

The superiority of UA, except ECRot, can be explained by the effect of acoustic cavitation, which generates waves capable of breaking the cell wall and increasing the antibacterial activity of irrigants ^17^. Previous studies have shown that ultrasonically activated sodium hypochlorite exposes more dentinal tubules and penetrates deeper than conventional irrigation ^8^ ^,^ ^18^. The results found by Li et al. ^19^ confirm our findings regarding the intratubular antibacterial effect of UA, which was superior to conventional irrigation in all root thirds. Additionally, in a recent proposal, Godoy et al. ^20^ evaluated the intratubular antibacterial action of photodynamic therapy and UA both associated or not, observing that UA has the greatest effectiveness regardless of whether associated. However, the use of an irrigant with antibacterial properties is important to achieve these results, as the physical action of UA alone does not lead to the highest percentage of damaged bacteria ^21^.

Although XPF and XPC are different in their shape and alloy heat treatment, these differences did not influence their intratubular antibacterial ability, as both were superior to conventional irrigation and inferior to UA. These results corroborate the findings of other authors who compared UA to a similar instrument (M3 Max) ^19^. In contrast, Pedrinha et al. ^22^ found no difference between UA and XPF, but the authors used saline solution during activation. These findings reinforce that only mechanical agitation of the irrigant inside the tubules is not enough to lead to a difference in the performance of the activators.

The bacterial culture resulted in fewer viable bacteria in the apical third. These findings can be explained by the lower density of dentinal tubules and the higher amount of sclerotic dentin in this region ^23^. This anatomical condition may not have influenced the microscopic analysis because the percentage of damaged bacteria was calculated based on the total number of bacteria (damaged and intact bacteria). The culture method used viable bacteria only as the count of the nonviable bacteria is not possible for this method. Azim et al. ^9^ found different microscopic results regarding thirds by using XPF and conventional irrigation. However, the authors limited the image field and set a depth up to 150 µm, and our study assessed the whole circumference of the root canal and had a depth of 300 µm. This factor should be considered, as different results can be obtained according to the analysis of the depth ^9^.

Although all activators had a positive performance, it should be taken into account that in vitro studies collect pure and standardized information without interference from variables, however, the clinical success is multifactorial. A recent systematic review also found that UA results in greater root canal disinfection than conventional irrigation ^24^. In contrast, Silva et al. ^25^ presented that UA has not better performance than conventional irrigation on periapical healing. These systematic reviews of the clinical trials suggest that although UA has more potent disinfection, it is not sufficient to make a difference in periapical healing. The present essay demonstrated the superior antibacterial ability of ECRot but no clinical studies of this activator on periapical healing have been carried out to date. Therefore, randomized clinical trials with this system are encouraged to confirm our results and verify the possible clinical relevance of this strategy on periapical healing. In addition, chemicals associated with sodium hypochlorite have been used during root canal preparation. The increase in dentin permeability caused by these associations may influence the intratubular antibacterial action, which also encourages future studies on this variable.

In view of the results and methodologies used, it can be concluded that the activation of the final irrigant enhances the disinfection of the root canal system, with ECRot having better results, followed by UA. ECRec, XPC, and XPF were similar but inferior to UA.
